# Study of Use of Products and Exposure-Related Behaviors (SUPERB): study design, methods, and demographic characteristics of cohorts

**DOI:** 10.1186/1476-069X-9-54

**Published:** 2010-08-29

**Authors:** Irva Hertz-Picciotto, Diana Cassady, Kiyoung Lee, Deborah H Bennett, Beate Ritz, Raea Vogt

**Affiliations:** 1 Department of Public Health Sciences, School of Medicine, University of California, Davis, One Shields Avenue, Davis, CA 95616, USA; 2 Department of Environmental Health, Graduate School of Public Health, Seoul National University, 748-220 Gwanak-Campus, 599 Gwanak-ro, Gwanak-gu, Seoul 151-742, South Korea; 3 Department of Epidemiology, School of Public Health, University of California, Los Angeles, 650 Charles Young Dr. South, Los Angeles, CA 90095-1772, USA

## Abstract

**Background:**

Exposure to toxic chemicals in the home is a growing concern. This report presents an overview of the recruitment, methods for data collection, instruments used to collect data, and participant demographics for a study examining behaviors that influence exposure to environmental toxins in the home environment, also known as SUPERB (Study of Use of Products and Exposure Related Behaviors).

**Methods:**

The methods involved three different platforms: telephone interviews, internet-based surveys, and home-based monitoring. The purposes of SUPERB were: first, to compare data collection platforms with regard to feasibility, acceptability and reliability; and second, to provide longitudinal population-based data characterizing seasonal and long-term changes in exposure-related behaviors including food consumption, temporal-spatial activity, and household product use.

**Results:**

Two cohorts of households were enrolled: families (one parent and one child) from northern California and older individuals (age 55+) from central California. Parents (n = 499) in Northern California families were on average 36 years of age, 47.1% were Latino or nonwhite, and 10.2% took the survey in Spanish. Most of the children enrolled (n = 566) were under 6 years (82.7%). The older adults enrolled (n = 156) were, on average, 66 years of age and 23.7% were Latino or nonwhite, but only 2.6% completed the survey in Spanish.

**Conclusions:**

We found that oversampling was successful in improving recruitment of under-represented subgroups, such as those with low education, thereby increasing diversity of our study sample. Protocols that minimize participant time, e.g., use of bar scanners and scales rather than questionnaires regarding use of household products, and the implementation of these protocols by staff who built relationships of trust, resulted in high retention over a longitudinal data collection scheme. A relatively small fraction of those who volunteer for longitudinal internet surveys are consistent in filling them out. Future reports will provide critical information on cross-sectional, seasonal and longitudinal patterns of exposure related behaviors in young children, parents of young children, and older adults.

## Background

The validity and precision of research on health effects that allows for quantitative risk assessment for environmental chemicals depend heavily on the quality of exposure information. Previous studies have been conducted to collect data on exposure to specific compounds for the U.S. population using large, probability samples [1,2]. These efforts have produced valuable information on group means and inter-individual variability in exposure levels. One lesson was that exposure to toxins is influenced by both micro-environmental levels of chemicals and by human activity patterns bringing persons in contact with exposures. Three types of human activity patterns are of particular interest for exposure assessment: food consumption, temporal-spatial activity patterns, and use of household products.

Food consumption serves as a source of exposures in several respects. First, it provides basic nutrient and caloric needs; second, it is a conduit for numerous chemicals that partition into the food during production that are not necessary for growth, development, or the maintenance of life systems; third, processing, packaging, preparation and cooking of food may introduce chemicals that were not present in the raw food. For some toxic compounds, food may be the primary route of exposure, e.g., to mercury, PCBs, some pesticides, dioxins, and acrylamide. While nutritional epidemiologists have focused on estimating nutrient and energy intake, food toxicology assesses contaminants and safe levels of essential minerals that are toxic at higher levels. A variety of methods are used for assessing dietary intake, reviewed by Kroes et al. [3], include food balance sheets, household market basket surveys, duplicate diet, food frequency, 24-hour recall, food records, and biomarkers. However, most large surveys providing extensive nutritional assessments, such as NHANES, are cross sectional providing limited insight into seasonal or long-term dietary behavior changes [4]. Although environmental contaminants of food has been a topic of increasing interest in recent years, especially among children, to date no surveys have comprehensively addressed exposure to non-nutritive chemicals through food.

Temporal-spatial patterns of activity strongly influence exposure in several ways. First, the specific micro-environment where people spend time influences exposure levels due to varying concentrations of an agent in that microenvironment. For instance, newer homes are constructed differently than older ones, with materials containing different chemicals. Second, certain types of human activities can increase exposure concentrations, such as smoking or burning candles, which causes higher ambient levels of polycyclic aromatic hydrocarbons and particles. Third, activities can influence the contact rate with the contaminated environment, for example, physical activity increases the total intake of a toxin through inhalation by increasing breathing rates. Important for children's exposures is crawling and hand-to-mouth activity, including placing their thumbs or other objects in their mouths, resulting in increased exposures via dermal or non-dietary ingestion, respectively. The National Human Activity Pattern Survey (NHAPS) [5] further generated activity pattern data intended for the estimation of the prevalence and duration of population exposure. Data from this survey are included in the U.S. Environmental Protection Agency's Consolidated Human Activity Database. However, previous studies concentrated on collecting short-term activity data, and have not emphasized longitudinal or seasonal data collection that would allow evaluation of intra-individual variability over time.

Finally, household products are a significant source of exposures to a variety of chemicals [6,7]. Products with direct dermal and inhalation exposures span a wide array of purposes: household cleaning products for furniture, toilet bowls, glass, carpets, etc.; pesticides (including those used on pets); dry cleaning fluids; pre-sale treatments of carpets and draperies for stain resistance and their backings with flame retardants; air fresheners, containing perfumes and aerosol propellants. Other household products relate to personal care including: cosmetics; dyes, permanents, hair straighteners, and shampoos; sunscreens; nail polishes; antiperspirants; perfumes and other fragrances used on the body or as room fresheners; contact lens solutions, ear wax removal products, and nasal sprays. Pesticides have been deemed of particular concern given that they may be formulated with the specific intent of having sufficient toxicity to kill certain species of living organisms. However, chemicals used for numerous other purposes also often have toxic properties. Very little research on use of household products has been conducted with the aim of understanding the potentially harmful human exposures that may result from their use.

A major gap in the current knowledge base concerning exposure assessment and human activity patterns derives from the cross-sectional or short-term nature of most population-based exposure datasets. The Study of Use of Products and Exposure-Related Behaviors (SUPERB) was developed in response to growing interest in data collection platforms that can be used for longitudinal assessments of exposure-related behaviors that may change over time, e.g., seasonally. The SUPERB platforms were designed to vary in the burden imposed on the participants and to be appropriate for several different age strata in population-based samples of households. Three age/demographic strata were of particular focus: young children, adults with families, and older adults. We conducted the surveys in northern and central California, the recruitment aimed to enroll an ethnically diverse sample. Data collected in a multi-tiered approach from SUPERB participants covered short-term, seasonal, and long-term changes in food consumption habits, temporal-spatial activity, and use of household and personal care products. It is expected that these data, as well as the methodological lessons learned, will be particularly helpful in refining protocols for studies such as The National Children's Study, the first comprehensive longitudinal assessment of a nationally representative sample of U.S. pregnant mothers and their children, aimed at examining exposures in early life and their health effects. The present report provides an overview of the SUPERB design and data collection methods and presents descriptive information on the study samples participating in various Tiers, each with a different platform or approach for data collection.

## Methods

### Study population and sampling

SUPERB aimed to ensure that several key periods of life would be well-represented but did not attempt to comprehensively include all life-stages. We sought to enroll a probability sample from two sampling frames, one consisting of households with young children (one adult and one child enrolled) and the other, of households with older adults (one older adult enrolled). Our goal was to recruit 550 households, of which two-thirds were families with two respondents (one parent, one child), and one-third with study participants over the age of 55. All recruitment and data collection protocols were approved by the institutional review boards at the University of California at Davis and Los Angeles.

**Figure 1:**
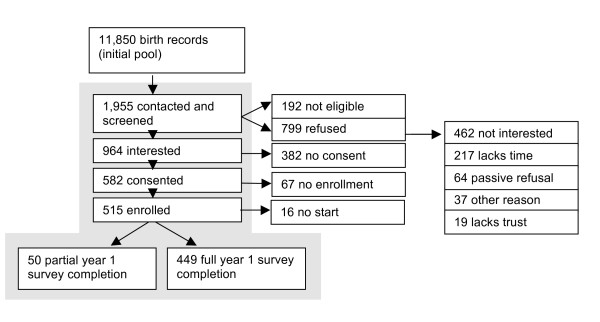
**Flow chart of recruitment and enrollment of *northern California families *into SUPERB (Study of Use of Products and Exposure-Related Behaviors)**.

Figure 1 shows the recruitment of households with young children that were sampled from files of California state birth certificate records [8]. Birth records were drawn from an 18-county region in northern California and were sampled randomly. The index child (the child on the birth certificate) was born between January 1, 2000 and December 31, 2005. Birth records were traced to determine contact information for parents listed on the birth record using several commercial databases/services. Once the phone numbers and addresses were obtained, two methods of recruitment were attempted: cold calls or introductory postcards sent prior to the telephone call. Altogether, we recruited from a pool of over 11,000 households sampled from the birth records. The selection of households occurred in six waves beginning 7/26/2005 through 1/10/2007, sampling was stratified to maternal education level (less than 12 years and 12 or more years) and the year of the child's birth, weighing the sample more heavily towards the group with lower education to compensate for an anticipated lower response rate from that group.

Once a family was reached by phone, eligibility was determined using the following criteria: a child under 6 years of age was living in the household and a parent spoke English or Spanish. We enrolled two members from each household: one parent and one child. Generally, the index child from the birth record was recruited into the study. The exception was the year 2006 when we targeted older children for recruitment to collect information on several age groups.

The second cohort was comprised of older adults randomly selected from residences identified from tax assessors' records using probability sampling by number of housing units. The sampling frame included residents in three counties in the central valley of California with high agricultural productivity, referred to as the cohort from central California. We used the following inclusion criteria: resident is 55 years of age or older; speaks English or Spanish; currently lives in Kern, Tulare, or Fresno counties; does not have Parkinson's disease.

**Figure 2:**
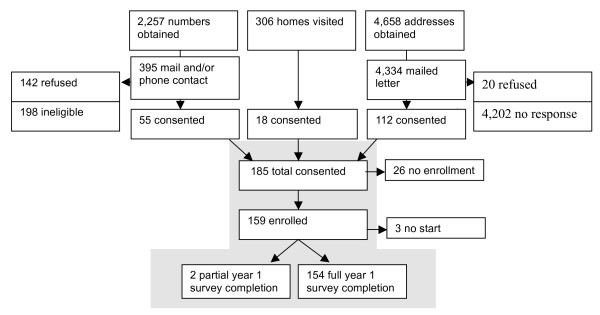
**Flow chart of recruitment and enrollment of *central California older adults *into SUPERB (Study of Use of Products and Exposure-Related Behaviors)**.

Three rounds of recruitment were conducted in central California to enroll older adults (Figure 2). In the first two rounds, we solely relied on address lists for residential parcels. For Tulare county, we obtained 1998 parcel maps and for Kern and Fresno counties those from the year 2001 from the respective county tax assessor's office. We randomly selected 500 parcels from all three counties six times and checked for duplicate and invalid records; excluding these yielded a total of 2,658 valid parcel addresses. We then attempted to recruit subjects by mail and phone. We obtained numbers for parcels from commercial services (Donnelly and Telematch) that whenever possible matched addresses to phone numbers (note that different from the birth certificate record information we did not have names for residents living at these addresses). All 2,658 parcels were submitted and 1,203 potential residents living at the selected addresses were received in return. We also conducted internet searches for addresses not matched to a phone number by the commercial services. Altogether, an attempt was made to reach approximately 2,257 households by phone. Recruitment letters were mailed to all addresses for which we were unable to get in contact with a person via telephone (n = 2,334). A large but unknown number of households were never reached by phone and might have been occupied by younger residents not eligible for our study. A total of 55 subjects were recruited through telephone contacts and 65 through mailings from the first round of recruitment.

For the second round of our recruitment, we sampled at one time an additional batch of 2,000 addresses, weighted according to each county's population size and mailed a screening questionnaire and a follow-up reminder. Households that returned the screening questionnaire indicating that they were possibly eligible and interested in participating were called to confirm eligibility (n = 47) and informed consent for participation was obtained at this time. Finally in a third round of recruitment we conducted door-to-door solicitations at 306 homes targeting previously randomly selected parcels and were able to enroll 18 additional participants. The total number of households with an eligible resident (i.e. parcels inhabited by a resident in the targeted age range speaking English or Spanish) among all parcels randomly sampled is unknown.

### Overview of data collection strategies and timing

**Figure 3:**
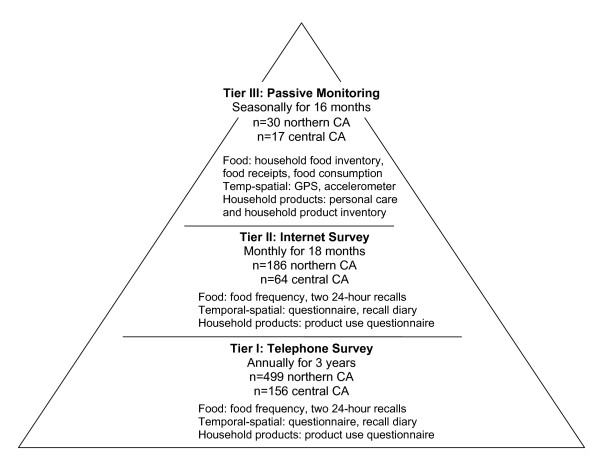
**Data collection platforms for thee tiers of SUPERB (Study of Use of Products and Exposure-Related Behaviors)**.

SUPERB collected data in three main Tiers corresponding to three platforms food consumption, temporal-spatial activity, and household products (Figure 3). The Tier 1 platform consists of an interviewer-administered questionnaire conducted via telephone for all participants enrolled in the study. All interviewers were extensively trained by senior staff to adhere to the protocols for both recruitment and administration of questionnaires to gather high quality data. A 40-page training manual guided trainees who practiced by conducting mock interviews, shadowing senior interviewers, and beginning to work under intense supervision; they started with recruitment calls, and after the first month they were allowed to move on to administering surveys. Of the 23 interviewers, 6 were college graduates and 17 were enrolled undergraduates at the University of California, Davis; 8 of the 23 were bilingual in Spanish.

The survey used questions from existing validated nutrition and activity surveys; new questions were created to cover an array of consumer and household products. New questions were also generated to capture specific dietary patterns, food preferences, other product uses, and occasionally-conducted activities. Tier I information was collected employing five telephone interview elements: one general survey collecting data about the past year, two 24-hour dietary recalls (weekend and weekday); and two 24-hour temporal-spatial activity recalls (weekend and weekday). Parents responded for themselves and for children under 8 years of age; children 8 years and older responded for themselves if they had their parent's permission. These telephone surveys were conducted in English or Spanish once each year for three years in a row to track changes in exposure-related behaviors. Because much of the telephone survey was based on previously validated instruments, standardized questionnaires, and/or commonplace data collection techniques, Tier I information serves as the reference for testing innovative data collection strategies used in Tiers II and III.

Tier II also captures longitudinal information, but on a more frequent basis, using the internet rather than telephone and a self-administered rather than interviewer-administered format. This internet survey was developed to evaluate the acceptability and reliability of a data collection platform that: (1) is significantly less expensive than a telephone survey, (2) provides the participant with more flexibility in choosing when to complete it, and (3) is relatively user-friendly and familiar to some [9]. Self-administered internet-based surveys mirrored as much as possible the questions asked in Tier I. To capture seasonal variability, participants were asked to complete surveys on a monthly basis for eighteen months, rotating through three instruments: (i) a general questionnaire focused on household products and food frequency, (ii) a weekday recall for 24-hour food consumption and temporal-spatial activity patterns; and (iii) a weekend recall for 24-hour food consumption and temporal-spatial activity patterns. If the three surveys were administered in March, April, May, respectively, then June repeated the survey administered in March, and so forth. In this manner, Tier II collected seasonal data on each of the behavioral domains for each subject with the goal of covering an 18 month time period.

Tier II enrolled a subset of households from Tier I. We targeted those who were able to complete the internet survey in English. Participants received periodic e-mail reminders about upcoming survey elements and a thank you e-mail was sent upon completion of each survey. In Tier II, parents respond for themselves and their children due to concerns about the amount of reading required in the internet surveys. For Tier I participants selected for Tier II who lacked a computer or internet service, we offered to provide the equipment, services, and an in-person orientation to the computer and the internet survey.

The third Tier was designed to require minimal time and effort from the participants in order to reduce the interview burden, increase retention, and thereby enhance generalizability of results. The platform utilized seven different passive measures of exposure-related behaviors. This data collection involved more intensive staff effort, however. We implemented Tier III with four monitoring intervals over a 16 month period, each one week long, in which staff visited the household at the start and end of the week. The passive measures to obtain food consumption data included: a video recording of meals and snacks prior to consumption for the entire week, collection of receipts of food purchases during the week, and a household inventory of certain foods collected at the beginning and end of the week [10]. To capture temporal-spatial activity, subjects were fitted with personal global positioning system (GPS) monitors and motion sensors which collected data throughout the week [11,12]. To gather household product use data, we inventoried certain classes of personal care and household care products at the beginning and end of the week and fitted the two most commonly used cleaning products with a motion sensor to record use of the product. By its nature, this platform did not provide individual-level data but rather household-level information.

### Data collection details for food consumption

#### Tier I: Telephone surveys of food consumption

In the annual telephone surveys, SUPERB obtained dietary behavior information using both 24-hour recalls and a food frequency questionnaire. We used the Continuing Study of Food Intake in Individuals (CSFII) as the protocol for the 24-hour dietary recall. The CSFII was previously reported to be reliable and valid when administered over the telephone [13]. In SUPERB, this survey was used to estimate exposure to toxics, as others have done [14,15]. Following the CSFII protocol, SUPERB participants, upon enrollment, were sent measuring cups and other materials to estimate portion sizes and for each interview were prompted with a series of standardized questions to assist their recall of the foods eaten during the previous day. Foods eaten were coded by trained interviewers using the USDA's Food and Nutrient Database for Dietary Studies (FNDDS). Questions on body metrics, dietary practices, and health status are included in the 24hour recall. To complement the recall, interviewers also administered a food frequency questionnaire (FFQ) asking about typical foods eaten in the last year [16]. The FFQ questions administered to children over the age of 7 were also adapted from "in the past year" to "in the past month" in anticipation that children may have greater difficulty recalling food consumption over the span of a year compared to their parents. For all of Tier I, the FFQ was also reduced from 126 to 44 food items to focus on key food groups associated with a higher risk of exposure to specific groups of toxics selected a priori: pesticides, metals, persistent halogenated compounds, acrylamide, hormones and antibiotics. Exposure to these compounds can be linked to public use datasets on contaminant levels in food. Questions about organic and canned food consumption, water bottle use, and food preparation/storage were added to the FFQ to further examine exposure to pesticides and xenoestrogens.

#### Tier II: Internet surveys of food consumption

In Tier II, the food frequency questionnaire was further reduced from 44 to 30 food items to shorten the time required to complete the survey. Structured questions were relatively easy to adapt to an internet-based survey administration format. In contrast to the food frequency questionnaire (FFQ), the 24-hour recalls required considerable programming and testing to generate the appropriate prompts for respondents, and to allow respondents to successfully find their foods in the USDA's Food and Nutrient Database for Dietary Studies.

#### Tier III: Passive measurement of food consumption

During the Tier III home visits, SUPERB field staff collected three sources of information to measure household food intake. First, foods eaten at home were captured by a motion-activated video camera system that was mounted above the kitchen counter. Participants were asked to place the food items on the counter prior to serving them to "record" the various meals. Food items were placed on placemats we provided, in order to indicate whether the plate of food was for the parent or child and to standardize portion sizes. The camera had no sound recording and did not record peoples' faces; it was left on for one week with motion activation. Second, the study staff completed a food inventory from the refrigerator, freezer and pantry during their visit at the beginning and end of the week. Foods included were those for which we elicited information during the interviews conducted in Tier I. Staff brought a laptop with a custom UPC tracking system application written in Hypertext Preprocessor (PHP) connected to a scale (OHAUS Model Scout Pro SP4001) and barcode scanner (Symbol LS2208) through USB connections. To input data, food items were placed on the scale. A publicly available database was searched for barcode data and if there was no product information available, product information was entered into the database. Fresh and bulk foods were tracked using study specific barcodes for categories of interest. Third, families collected food receipts from all sources (supermarkets, restaurants) for one week. In addition, they noted if they lost a receipt or acquired food without a receipt, such as eating at work or a friend's house.

After collecting these sources of data for Tier III, we created variables suitable for analysis for a final dataset containing FNDDS food codes for every food item and the amounts of nutrients/compounds in those foods. For instance, standard methods for content analysis of media were used to code and develop a dataset from the video tape [17]. Staff listed foods from video images on a form similar to 24-hour dietary recalls from Tier I, then coded foods and amounts using the FNDDS foods database. For the food inventory, UPC codes were linked to FNDDS food codes. For food receipts, field staff entered the item, quantity, and price into a database developed for a previous study by other researchers [18].

### Data collection for temporal-spatial activity patterns

#### Tier I: Telephone surveys of temporal-spatial activity

In the first year, participants were asked to complete two 48-hour recall surveys reflecting activities during the weekdays and the weekend. Previous recall studies have included only a 24-hour period of recall [19] but we wanted to determine if reliable information could be obtained for the second 24-hour period. We collected 24-hour recalls only in the second and third years, in the interest of time. The 24-hour interview consisted of guiding the participant through their day beginning at midnight and following through to midnight. The participant first recorded time spent at each location, then the interviewer reviewed the survey with the participant to ensure that all times and locations were captured correctly. The location categories used were compiled from the NHAPS data [5] and the California Activity Patterns Surveys [20] and then adapted for our purposes. The following locations were coded in year 1 (with slight modification in year 2): residential locations including home, garage, or someone else's home; school/childcare locations; places for work, shopping, and eating i.e. office buildings, stores, restaurants, and service locations; and other locations such as public buildings, religious institutions, and recreational facilities. Additional questions were asked about activities that occurred less frequently (barbeque, pumping gas, going to a bar/night club, etc.). Various modes of transportation were also included when eliciting time spent in transit. The focus was on locations and therefore only minimal information on activity was obtained, e.g., sleeping, working, and awake not working. Some of the specified activities are associated with exposure to a particular compound, for instance, polycyclic aromatic hydrocarbons and particulate matter while barbequing or benzene and other volatile organic compounds while pumping gas. Concentration distributions have been measured in some of the various microenvironments in other studies and can be used in models to estimate exposures.

#### Tier II: Internet surveys of temporal-spatial activity

The participant completed the internet survey in one of every three months, covering both a weekday and a weekend day. Similar to the temporal-spatial telephone interview, the Tier II 24-hour diary was to determine the amount of time spent in different types of activities and locations. Location categories (same for Tier II as Tier I) and the corresponding time spent at each location were recorded with an internet interface that allowed participants to create blocks of time spent in each location. Participants found this method cumbersome and so a second interface was developed for the later part of the study that traded flexibility for simplicity. The second interface required that the participant go through their day in a linear fashion rather i.e. in the order in which they occurred. Both interfaces use flash technology™ using Adobe Flash Player. This platform was selected from available internet devices because it was supported by most modern operating systems and had very minimal hardware requirements.

#### Tier III: Passive monitoring of location and physical activity

In Tier III, participants were asked to wear a GPS attached to their waist in a small pouch, similar to the pouches used to carry a mobile phone. The GPS we selected (Skytrx minitracker MT4100) was found to have spatial accuracy within 2.5 meters outdoors. The GPS did not record indoors, and the gaps therefore allowed us to quantify time spent indoors. The unit has a USB port and data were downloaded onto a computer by study personnel during their field visits and processed using Skytech software that interfaced with the internet, providing both the actual GPS locations at each time and a record of times spent at each residential block. The observation period was one week and participants were asked to wear it upon waking. Time resolution was every second, sufficient to capture the movement of subjects outdoors.

Participants were also asked to wear two accelerometer activity monitors, one attached to the GPS unit worn on the right hip while awake and the second on the wrist, to be worn at all times. The sensor co-located with the GPS also allowed us to determine the compliance with wearing the GPS device. If the accelerometer recorded no movements, it was unlikely that it was being worn. We selected the Actical Accelerometer which contains a biaxial piezoelectric accelerometer sensor to record physical motion in two planes. Regression equations have been developed for these devices when worn on the hip to relate the number of movements of the participants' metabolic equivalent task (MET) levels [21].

For most of the participants, we asked them to record the places they visited during the final week they were wearing the GPS and accelerometer. We will use these data along with the GPS data to determine the coordinates of the places they visited that week. The synchronized GPS and activity data are the basis for determining temporal-spatial activity pattern in our study participants.

It was not practical to have young children wear the GPS device on their waist. Thus, children were asked only to wear one accelerometer on their wrist. While their data cannot be used to convert to quantified MET data, we will document variability and categorize activity levels accordingly.

### Data collection for household and consumer product use

To date, the research community has not developed generally accepted standardized and validated instruments 'for the collection of household and personal care product use, though numerous research organizations have developed instruments. Since there are no standards for these methods, we developed some questions based on the experiences of our investigators and staff whenever adequate existing questions were not identified.

#### Tier I: Telephone surveys of household & personal care product use

The telephone interview was developed and then evaluated during the pilot phase we conducted in the first year. During pilot data collection, participants were prompted with categories of frequency of usage. Since some products are used quite rarely (e.g., oven cleaners) and others on a day-to-day basis (e.g., cosmetics, antiperspirants), the instrument allowed participants to state the frequency and select the units of time (i.e., per day, week, month, year, etc.) to capture a wide range of possible frequencies of use and thereby enhance the informativeness of responses. Factors that might mitigate exposures (e.g., use of gloves or other protective gear, or keeping the child away from a sprayed area following pesticide applications) were also queried referring to the most recent use of the product. All questions pertained to the respondent, or the enrolled child in households with children. Product categories were selected to capture use of products likely to contain 1,4-dichlorobenzene/Naphthalene, adhesives, solvents, ammonia, benzene, pyrethroid pesticides, disinfectants, mercury, and phthalates.

#### Tier II: Internet surveys of household & personal care product use

The internet survey mimicked the annual interview; however, the time frame for most of the questions was different. In Tier I, questions asked to report "the last time", or "within the past year", or "how often do you". The goal of Tier II was to capture seasonal variation, and as described above, the same questionnaire was repeated every three months. Thus the per-year questions were changed to refer to the past three months instead.

#### Tier III: Passive monitoring of household & personal care product use

In Tier III, an inventory of household and personal care products was conducted at the start and end of a one-week period, seasonally. We pre-selected a number of categories of household cleaning, pest control, personal care, and other products. The type of item and their weights were tracked employing the same method as used for food products. In addition, for ease of recording and classification, we created study item generated barcodes, one for each product type of interest (i.e. body lotion, liquid soap). We scanned the appropriate barcode on the list and thus easily classified the product into its respective class.

The system also allowed us to record whether or not the specific product container had been seen at a prior visit, allowing us to track specific containers and their use over time. Using the weights of these containers from both the start-of-week and end-of-week visits, we determined a weekly change in mass for each product class. If a given product was not found on both visits, change in mass could not be determined. The participant was also asked to identify the two cleaning products they used most frequently. An Actical accelerometer was strapped to each of the two products to record how often this product was moved during the week.

## Results

The SUPERB sample consisted of 499 northern California families and 156 central California residents, with the original enrollment goal set at 500 families with young children and 250 older adults enrolled in Tier 1 (see Table 1). Enrollment in Tier II was conducted by recruiting from among eligible participants in Tier I. The target was to enroll a total of 250 households and the final number enrolled was 186 northern California and 64 central California households. For Tier III, we also recruited from Tier I participants who were not already participating in Tier II; our goal was a total of 40 households. We exceeded our goal, with the final tally of 47, of which 30 households were from northern, and 17 from central California.

**Table 1:** Enrollment and completion rates of Tiers I, II, and III

	Targeted enrollment; (% recruited northern, central)	Actual enrollment (northern, central)	Full data completion (northern, central)	Only partial data completion (northern, central)	Only minimal data completion (northern, central)
Tier I: year 1 only			5 survey elements	3-4 survey elements	1-2 survey elements
	n = 750	n = 655	n = 580	n = 31	n = 39
	(26%,32%)	(499, 156)	(87%,95%)	(6%,2%)	(7%,3%)

Tier I: longitudinal (years 1-3)			3 years	2 years	≤1 year
	See above	See above	n = 252	n = 182	n = 221
			(38%,41%)	(25%,37%)	(37%,22%)

Tier II: 15/18 months^a^			15-18 monthly surveys	10-14 monthly surveys	1-9 monthly surveys
	n = 250	n = 250	95^b^	26	129
	(77%,75%)	(186, 64)	(29%,63%)	(11%,9%)	(60%,28%)

Tier III: 8 visits over 4 seasons^b^			4 collection periods^c^	2-3 collection periods	1 collection period
	n = 40	n = 47	n = 40	n = 4	n = 3
	(34%,52%)	(30, 17)	(90%,76%)	(7%,12%)	(3%,12%)

^a ^Full completion depended upon timing of enrollment into tier 2 as it was modified from 15 to 18 months.

^b ^Includes participants (n = 53) who attempted to complete surveys but encountered issues with the internet survey.

^c ^Each collection consisted of a set-up visit and a take-down visit at the beginning and end of a week. The goal was for four collection periods across a 16 month period.

The enrollment rate for Tier I was 25.5%, enrolling 499 out of 1,955 families contacted (Figure 1). The most common reason given for refusal was lack of interest (n = 462) followed by lack of time (n = 217), passive refusal (never said no, but never said yes, n = 64), an unstated reason (n = 37), and lack of trust (n = 19). In central California, of 527 older individuals reached by mail (n = 132), or both mail and phone (n = 395), 167 (31.7%) enrolled in Tier I of the study (this figure does not include the non-random home visits during which an additional 18 consented and an unknown number of those actually enrolled). There were higher enrollment rates were for Tier II at 77% and 75% for northern and central California, respectively, compared to enrollment rates for Tiers III of 32% and 52%, both of which were recruited from Tier I. However, Tier II also had the lowest retention rates, with 64% and 28% of northern and central California, respectively, not fully completing the survey instruments compared to 3-12% for the other Tiers. The first year of Tier I surveys, during which we conducted 5 surveys over approximately one month, had the highest overall completion rate (87% and 95% for northern and central CA) but these rates dropped in the longitudinal data collection in which the full completion rate (all 3 years of surveys) was 38% and 41% respectively. In Tiers II and III, completion rates were mixed. As mentioned above, Tier II had the lowest full completion rates compared to other Tiers but rates differed between cohorts with older adults having a much higher full data completion rate (63% vs. 29%). Tier III had full data completion rates (4 visits over 16 months), of 90% and 76% for northern and central California participants, respectively, nearly as high as Tier I first year data collection.

Table 2 provides a description of contaminants we considered when deriving relevant questions for the survey instruments. Thirteen categories of compounds were targeted for investigation based on their widespread use and high likelihood of being a source of contamination in the home environment. Data on exposure to these compounds was collected via seven categories including: residential information, food frequency, 24-hour food recall, supplemental food questions, temporal-spatial activity questions, consumer products, and personal care products. For example, pesticide information came from the food frequencies and 24-hour food recalls (fruits and vegetables high in pesticide residues), household product use (sprays or foggers used indoors or outdoors), and personal care product use (insect repellant).

**Table 2:** Description of contaminants in the home environment included in the study

Compound of concern	Sources/exposures considered	Questions	Residential Information	Food recall/frequency	Food Questions	Time activity	Consumer products	Personal care products
1,4-Dichloro-benzene/Napthalene	Toilet bowl and other solid deoderizers	Use of toilet bowl and other solid deoderizers and moth repellents					x	

Arcylamide	High temperature baked goods	Consumption of carbohydrate-based foods		x				

Adhesives and solvents	Various BTEX components	Hobbies and exposure to cleaners, compounds; recent home improvements	x			x	x	

Ammonia	Household cleaners	Use of various household cleaning products					x	

Antibiotics and hormones	Animal food	Meat, dairy, egg, poultry, and fish consumption		x				

Benzene	Cigarette smoke, gasoline	Smoke exposure; transportation; gas storage; proximity to gas station, busy road	x			x	x	

Current use pesticides (pyrethroids)	Food, residential use, agricultural exposure	Fruit, vegetable, and organic food consumption; residential pesticide and insect repellent use	x	x	x	x		x

D-Limonene and α-Pinenene	Lemon/pine scents	Air freshener, household cleaner use					x	

Disinfectants	Household cleaners	Household cleaners, air fresheners, antibacterial soaps					x	x

Mercury	Fish, various products containing mercury	Fish and shellfish consumption; use of nasal sprays, sunscreens, hair dyes, contact lens solutions, and ear drops		x				x

PAHs, HAAs^a^	Charcoal	Proximity to outdoor BBQ				x		
Phthalates	Water bottles, food containers; Personal care products	Use of plastic food containers, hair products, nail polish, lotions, deodorants, and cosmetics			x			x

TCE	Dry cleaning	Use of and proximity to dry cleaners	x				x	

^*a*^*PAHs (polycyclic aromatic hydrocarbons), HAAs (heterocyclic aromatic amines);*

category also includes particulate matter, carbon monoxide, VOCs.

Table 3 shows demographic characteristics collected on enrolled subjects. From both northern and central California, the adult respondents were primarily females (data from Tier I interviews): >80% females in Tiers I and II from households with young children, and about two-thirds from households of persons over 55 years of age. The Tier I sample was 47% Latino or nonwhite in the northern California households of families with young children, and 24% Latino or nonwhite in the central California sample of older adults. In Tier II, these figures were 34% and 14% in northern and central California, respectively, and in Tier III, 23% and 31%. Thus, the trend was reversed, such that nonwhite families were progressively less likely to participate when moving from Tier I to II to III in the Northern California cohort, whereas diversity of older adults was highest in Tier III. In northern California, employed persons comprised around 50% of participants in Tiers I and II, this dropped to 35% in Tier III; among older adults in central California, the figures for employed persons were 40% in Tiers I and II and this dropped to 25% for Tier III. In both age groups/regions, the percentage married was higher for Tier II than for Tier I or III. Place of birth (U.S. vs. abroad) did not differ dramatically across the Tiers. In the first year of Tier I data collection, 10.2% of northern California participants took the survey in Spanish, as compared with 2.6% of the central California older adults. Similar to the northern California cohort, the central California sample represented a relatively privileged group- the majority being home owners (86.6%) compared to a home ownership rate of 53.8% among the general population of the (combined data from included central California counties, data not shown) [22]. Among minors enrolled (n = 566, we sometimes enrolled two per household), the age distribution reflected the emphasis we placed on the early life stage primarily recruiting from among early life stages: early childhood (<6 years): n = 468 (82.7%), middle childhood: (6-12 years) n = 77 (13.6%) and adolescence (12- 17 years): n = 21 (3.7%) (data not shown).

**Table 3:** Demographic characteristics, as reported by respondent during interview, for the northern and central California cohorts

	Northern California Family Cohort			Central California Older Adult Cohort		
	**Tier 1**^**a**^	**Tier II**	**Tier III**	**Tier I**^**b**^	**Tier II**	**Tier III**

**Households (n)**	499	186	30	156	64	17

**Mean age**	37	35.7	34.7	66	63.8	68.6

**Female age group (n)**	412	157	28	102	41	13
18-34 years	35.0%	37.6%	32.1%	n/a	n/a	n/a
35-54 years	57.3%	61.8%	53.6%	2.9%	4.9%	n/a
55-64 years	0.5%	0.6%	0.0%	47.1%	58.5%	30.8%
65+ years	0.0%	0.0%	0.0%	49.0%	36.6%	53.8%
Missing	7.3%	0.0%	14.3%	1.0%	0.0%	15.4%

**Male age group (n)**	87	29	2	54	23	4

18-34 years	24.1%	34.5%	50.0%	n/a	n/a	n/a
35-54 years	65.5%	62.1%	50.0%	n/a	n/a	n/a
55-64 years	2.3%	3.4%	0.0%	55.6%	56.5%	75.0%
65+ years	0.0%	0.0%	0.0%	40.7%	43.5%	25.0%
Missing	8.0%	0.0%	0.0%	3.7%	0.0%	0.0%

**Adult sex**	499	186	30	156	64	17

Female	82.6%	84.4%	93.3%	65.4%	64.1%	76.5%
Male	17.4%	15.6%	6.7%	34.6%	35.9%	23.5%
Missing	0.0%	0.0%	0.0%	0.6%	0.0%	0.0%

**Female education level (n)**	412	157	28	102	41	13

0 -11 years	7.3%	1.3%	3.6%	3.9%	0.0%	7.7%
12 years	12.9%	8.3%	7.1%	16.7%	7.3%	7.7%
13-15 years	23.3%	28.7%	14.3%	40.2%	46.3%	38.5%
16 years	28.2%	31.2%	39.3%	18.6%	24.4%	23.1%
>16 years	20.9%	30.6%	21.4%	18.6%	22.0%	7.7%
Missing	7.5%	0.0%	14.3%	2.0%	0.0%	15.4%

**Male education level (n)**	87	29	2	54	23	4

0-11 years	3.4%	10.3%	50.0%	3.7%	0.0%	0.0%
12 years	10.3%	17.2%	0.0%	3.7%	0.0%	0.0%
13-15 years	19.5%	31.0%	50.0%	24.1%	21.7%	25.0%
16 years	26.4%	41.4%	0.0%	37.0%	43.5%	50.0%
>16 years	32.2%	0.0%	0.0%	25.9%	34.8%	25.0%
Missing	8.0%	0.0%	0.0%	5.6%	0.0%	0.0%

**Race/ethnicity (n)**	499	186	30	156	64	17

Non-Latino white	50.5%	63.4%	73.3%	76.3%	84.4%	52.9%
Latino (of any race)	20.5%	12.9%	20.0%	10.3%	7.8%	29.4%
African American	2.8%	2.2%	0.0%	1.3%	0.0%	0.0%
Asian	10.8%	9.7%	3.3%	0.0%	0.0%	0.0%
Other	5.0%	7.5%	0.0%	6.4%	4.7%	5.9%
Multiple	2.8%	4.3%	0.0%	3.2%	3.1%	5.9%
Missing	7.6%	0.0%	3.3%	2.6%	0.0%	5.9%

**Employment status (n)**	499	186	30	156	64	17

Retired	0.2%	0.5%	0.0%	46.2%	46.9%	52.9%
Employed	46.9%	54.8%	30.0%	37.8%	42.2%	23.5%
Stay at home parent	35.1%	36.0%	53.3%	2.6%	0.0%	5.9%
Unemployed	2.4%	1.1%	3.3%	5.8%	6.3%	0.0%
Other	7.6%	7.5%	0.0%	5.1%	4.7%	5.9%
Missing	7.8%	0.0%	13.3%	2.6%	0.0%	11.8%

**Foreign born (n)**	499	186	30	156	64	17

Yes	28.5%	22.6%	20.0%	8.3%	4.7%	5.9%
No	64.1%	77.4%	66.7%	89.1%	95.3%	82.4%
Missing	7.4%	0.0%	13.3%	2.6%	0.0%	11.8%

**Homeowner (n)**	499	186	30	156	64	17

Yes	70.1%	78.5%	73.3%	87.2%	92.2%	76.5%
No	22.2%	21.5%	13.3%	10.3%	7.8%	11.8%
Missing	7.6%	0.0%	13.3%	2.6%	0.0%	11.8%

**Marital status (n)**	499	186	30	156	64	17

Married/living together	87.2%	95.7%	80.0%	57.7%	68.8%	47.1%
Widowed	0.0%	0.0%	0.0%	21.2%	12.5%	23.5%
Divorced/separated	1.6%	1.1%	3.3%	13.5%	15.6%	11.8%
Single	3.4%	3.2%	3.3%	5.1%	3.1%	5.9%
Missing	7.8%	0.0%	13.3%	2.6%	0.0%	11.8%

**Survey in Spanish (n)**	499	186	30	156	64	17

Yes	10.2%	1.1%	10.0%	2.6%	0.0%	5.9%
No	82.6%	98.9%	76.7%	95.5%	100.0%	88.2%
Missing	7.2%	0.0%	13.3%	1.9%	0.0%	5.9%

^a^Of the 499 northern California households enrolled, 462 completed a first-year demographic interview.

^b^Of the 156 central California households enrolled, 153 completed a first-year demographic interview.

In comparison with the target population for Tier I northern California families, those who participated in the study were significantly older, more educated, and less likely to have had a publicly funded delivery (Table 4).

**Table 4:** Maternal demographic characteristics recorded on birth certificates of northern California samples^a^

	Northern California Family Cohort				
	**Source population**	**Contacted**	**Tier I**	**Tier II**	**Tier III**

**Number of households**	11,850	1,955	499	186	30

**Age**		*^b^	*^b^		

Mean	28.7	30.8	31.6	31.7	30.3

**Age group**		*^b^	*^b^		

Under 25 years	27.8%	17.2%	13.4%	11.3%	16.7%
25-34 years	52.4%	53.9%	53.3%	58.1%	56.7%
35-44 years	19.5%	28.3%	33.1%	30.1%	26.7%
45+	0.3%	0.6%	0.2%	0.5%	0.0%

**Education level**		*^b^	*^b^	**^c^	

0-8 years	8.3%	7.2%	4.6%	0.5%	0.0%
9-11 years	12.8%	10.4%	6.4%	1.6%	6.7%
12 years	25.9%	16.3%	12.6%	12.4%	10.0%
13-15 years	19.9%	20.2%	21.0%	22.0%	10.0%
16 years	17.7%	23.7%	24.6%	26.3%	33.3%
>16 years	15.4%	22.3%	30.7%	37.1%	40.0%

**Race/ethnicity**^**d**^		*^b^	*^b^		

White	71.0%	71.3%	80.0%	79.6%	93.3%
Asian	20.2%	23.6%	15.6%	16.2%	6.7%
African American	6.5%	3.5%	3.6%	3.2%	0.0%
Native American	0.7%	0.3%	0.2%	0.5%	0.0%
Other/Missing	1.6%	1.3%	0.6%	0.5%	0.0%

**Hispanic identification**		*^b^	*^b^	**^c^	

Latino	33.2%	24.1%	21.6%	11.9%	23.1%
Non-Latino	66.5%	75.6%	78.2%	87.6%	76.9%
Missing	0.3%	0.3%	0.2%	0.5%	0.0%

**Place of birth**		*^b^	*^b^	**^c^	

USA	55.3%	56.8%	68.5%	76.3%	80.0%
Mexico	18.4%	13.9%	10.6%	2.7%	6.7%
Other	26.2%	29.2%	20.6%	21.0%	13.3%
Missing	0.1%	0.1%	0.2%	0.0%	0.0%

**Publicly funded delivery**		*^b^	*^b^		

Yes	33.6%	21.5%	17.0%	8.6%	13.3%
No	66.2%	78.4%	82.6%	91.4%	86.7%
Missing	0.2%	0.2%	0.4%	0.0%	0.0%

^a^All households participated in Tier I: some also participated in either Tier II or Tier III. Data shown are column percents.

^b^P value of comparison source population. ^c^P Value of comparison to Tier 1 cohort.

^d^Asian ethnicity includes Indian and Filipino. Native American includes Hawaiian, Aleut, Eskimo.

* Indicates significance at the <0.0001 level.

** Indicates significance at the 0.0001 level.

The majority of SUPERB participants possessed a computer and an internet connection at the start of the study. Among Tier I families (n = 499), 84.0% (n = 419) owned a computer and 79.8% (n = 398) had internet service. Among the older adults in the study sample (n = 156), 78.9% (n = 123) had a computer and 73.7% (n = 115) had internet service (data not shown).

## Discussion

SUPERB has enrolled participants from over 500 households and collected detailed data on exposure-related behaviors, using several platforms for persons at three life stages. Here we presented data on recruitment and enrollment and data collection methods along with demographic characteristics of participants. Future analyses will provide information on the accuracy, precision, cost, feasibility, and user acceptability of the three different platforms with respect to longitudinal data on food consumption, temporal-spatial activity, and household product use. Recruitment and retention of a culturally and socioeconomically diverse sample in a longitudinal study is challenging. For our northern California sample, we succeeded in enrolling households across the range of education, but many families could not be traced, particularly when the mother was younger. The resulting study sample, based on the more traceable and less mobile, was somewhat less diverse than our target population, although it did appear that oversampling of those in the lowest education groups was helpful. Similarly with central California older adults, our enrolled population consisted of a less diverse sample than the general population. As in all studies, enrolling those lower on the socioeconomic scale, who in general are disproportionately exposed to toxics compared to wealthier populations [23], raises challenges. Nevertheless, Tier I and to a lesser extent, the other Tiers, did include participants from all race, ethnic, and education groups, and hence, we expect our results to be valuable in understanding how best to measure exposure-related behavior at the individual and household level throughout the population.

Retention in the study varied across Tiers and cohorts in both intuitive and surprising ways. The first year of surveys issued through Tier I had the highest data completion rates, as would be expected from newly-enrolled, willing participants. Completion of the telephone surveys dropped off over three years though third year completion rates are lower than the first two partly because not all families were approached for the third year, as a result of time constraints. Among California families that also enrolled in Tier II and that therefore were asked to complete monthly internet surveys over 18 months in addition to the Tier I annual survey, fewer than one-third completed all or nearly all of them. It was surprising to see a retention rate twice as high (63% vs. 29%) for internet surveys among older adults (mean age = 64 years) than for parents (mean age = 36 years). We may infer that the limited free time among parents of young children is a stronger factor in influencing retention than the technological divide that one might expect to be an impediment for older adults. However, retention might be lower in a population of older adults with less education.

Consistent with our hypothesis that the time burden was a prime determinant of completion rates, Tier III, which had the lowest burden of participation (least amount of time required), had the highest longitudinal retention for northern California families: fully 90% of those who agreed to be in the home visit protocol, completed all eight visits. We also found that enrollment rates were not necessarily reflected in retention rates. While it was relatively difficult to enroll participants in Tier I, once enrolled, participants were willing to further enroll in other Tiers--more so for Tier II than Tier III--perhaps because of the more intrusive nature of Tier III. However, once enrolled in Tier III, participants had higher compliance than those in Tier II.

Future analysis will provide critical information on the cross-sectional, seasonal and longitudinal patterns of exposure related behaviors in young children, parents of young children, and older adults. Where feasible, the behavioral data will be applied to estimate concomitant exposures from environmental sources inside homes; this information can ultimately provide input for the design of effective programs geared toward education aimed to reduce exposure to toxins in the home environment. Finally, the SUPERB database will be made available for public use in a format compatible with the Consolidated Human Activity Database.

## Conclusions

Results can be used for planning future studies and streamlining ongoing studies. For researchers who wish to maximize compliance and retention in longitudinal studies, SUPERB methods and data collection platforms provide clear lessons. First, we found that oversampling of difficult to reach populations can be effective in leading to a more diverse sample than otherwise might be recruited. Secondly, we were able to achieve a remarkably high retention rate using a home visit protocol that minimized participant burden (time). Another factor in the high retention may also have been the deployment of the same study staffpersons to visit the households every time, resulting in a high level of trust. We therefore recommend that future studies be conscientious of the participants' time, particularly for families with young children, and build trusting relationships with participants to improve retention. Thirdly, while participants will volunteer for internet surveys, they are not always consistent over time, in completing them. Finally, these platforms and methodologies show promise for being useful in the National Children's Study or in other longitudinal investigations concerned with assessing environmental chemical exposures and their possible health effects.

## Abbreviations

US EPA: United States Environmental Protection Agency; SUPERB: Study of Use of Products and Exposure Related Behaviors; GPS: global positioning system; CSFII: Continuing Study of Food Intake in Individuals; FNDDS: Food and Nutrient Database for Dietary Studies; FFQ: food frequency questionnaire; PHP: Hypertext Preprocessor; MET: metabolic equivalent task; STAR: Science to Achieve Results

## Competing interests

The authors declare that they have no competing interests.

## Authors' contributions

IHP was responsible for conception, acquisition of funding, and general supervision of the research group. IHP, DC, KL, DB, and BR made substantial contributions to data collection and study design. IHP, DC, DB, and RV contributed to analysis and interpretation of data; IHP, DC, KL, DB, BR, and RV were involved in drafting the manuscript or revising it critically for intellectual content. All authors read and approved the final manuscript.
